# Dietary intakes of total polyphenol and its subclasses in association with the incidence of chronic kidney diseases: a prospective population‐based cohort study

**DOI:** 10.1186/s12882-021-02286-1

**Published:** 2021-03-10

**Authors:** Parvin Mirmiran, Emad Yuzbashian, Pegah Rahbarinejad, Golaleh Asghari, Fereidoun Azizi

**Affiliations:** 1 Nutrition and Endocrine Research Center, Research Institute for Endocrine Sciences, Shahid Beheshti University of Medical Sciences, 19395 − 4763, Tehran, Iran; 2 Endocrine Research Center, Research Institute for Endocrine Sciences, Shahid Beheshti University of Medical Sciences, Tehran, Iran

**Keywords:** Polyphenols, Glomerular Filtration rate (GFR), Chronic kidney disease (CKD), Tehran lipid and glucose study (TLGS)

## Abstract

**Background:**

As chronic kidney disease (CKD) is amongst the current global health challenges, this study is aiming to evaluate the long-term intake of total polyphenol and its subclasses in association with CKD incidence.

**Methods:**

For the purpose of this study, a sample of 3021 Iranian adults (47 % men, aged 20–79 years) with no CKD diagnosis at baseline, were selected from the Tehran Lipid and Glucose Study population. The total intake of polyphenol and its major subclasses were assessed by a validated food frequency questionnaire and categorized as flavonoids, phenolic acids, stilbenes, and lignans. Although the morphological abnormalities of the kidneys or 3-month persistent urinalysis can distinctively define CKD, the glomerular filtration rate (eGFR) reduction is accepted as a more precise index of renal function. Therefore, eGFR < 60 mL/min/1.73m^2^ was the exclusive index of CKD diagnosis in the current study. The eGFR was calculated by the Modification of Diet in Renal Disease Study equation. Cox-regression analysis was used to assess the hazard ratio and 95 % confidence intervals of CKD in quartiles of the total polyphenols.

**Results:**

In this study, 355 CKD cases over 11,058.464 person-years was reported. The median (IQR) age of participants was 36 years (27–46) at baseline. Moderate intake of lignans (≤ 6.8 mg) was negatively associated with the incidence of CKD in the adjusted model. No significant associations were detected between higher amounts of lignin and total polyphenols (HR: 0.97, 95 % CI 0.67–1.40) and CKD incidence.

**Conclusions:**

Based on the current findings, moderate intake of lignin possess CKD-protective properties by approximately 32 %. No independent associations were observed between higher amounts of lignins and CKD incidence.

## Background

Chronic kidney disease (CKD) is a public health challenge worldwide. It is commonly manifested by the loss of function or structural damage to the kidneys [1, 2]. Obesity, diabetes, hypertension and increased levels of inflammation and oxidative stress are the common risk factors of cardiovascular disease (CVD) and CKD [1, 3]. As habitual dietary intake is an effective modifiable factor in the CKD etiology, adopting a healthy lifestyle can prevent the incidence of CKD [4].

Adherence to specific dietary patterns including the Mediterranean diet [5] and the Dietary Approaches to Stop Hypertension (DASH) [6, 7] can positively contribute to the renal function. Alternatively, plant-based diets with higher constitution of fruits, vegetables, nuts, spices, herbs, cereals, legume, seeds, chocolate, and tea can prevent the occurrence of chronic conditions [8]. Plant-based food items are enriched with a diverse content of phytochemicals [9]. Polyphenols are the major micronutrients constituent of human diet, which are involved in many antioxidant and biological activities and affect various chronic disorders [10, 11]. Flavonoids, phenolic acids, stilbenes, and lignans are the four major subclasses of polyphenols [8]. Several studies have previously investigated the effect of polyphenol-rich red grape juice [12], green tee, coca [13] and white wine [14] in patients with renal failure. However, the CKD-preventive property of polyphenols in a regular diet is yet a matter of ongoing debate. Given the lack of adequate data on adults, this study aimed to investigate the association between long-term consumption of total polyphenol and its subclasses, including flavonoids, phenolic acids, stilbenes, and lignans with the incidence of CKD.

## Methods

### Study population

This longitudinal study was performed within the framework of the Tehran Lipid and Glucose Study (TLGS). This prospective study was launched in 1999 in five phases and primarily aimed to address and prevent the non-communicable diseases’ (NCDs) risk factors [15]. In spite of the first phase with a cross-sectional design, the four subsequent phases (II: 2002–2005, III: 2006–2008, IV: 2009–2011 and V: 2012–2015) were performed as prospective follow-up surveys.

The current study, a representative sample of 3021 individuals from phase III with 20–79 years of age and complete data, were recruited and followed-up. Pregnancy and lactation, energy consumption (800 > x > 4200 kcal/d), specific dietary patterns and any history of myocardial infarction, cerebral vascular accident or cancer at baseline was considered as the exclusion criteria. Ultimately, 2054 individuals were enrolled for the final follow-up analysis of 5.4 years.

All participants were initially asked to provide written informed consent.The study protocol was also approved by the ethics committee research council of the Research Institute for Endocrine Science (RIES), Shahid Beheshti University of Medical Science, Tehran, Iran.

### Dietary assessment

The habitual dietary intake was evaluated by a valid and reliable semi-quantitative food-frequency questionnaire (FFQ) at baseline [16, 17]. The individual consumption frequency of each food item was designated by trained and experienced dietitians on daily, weekly or monthly basis. The portion sizes were collected in household measures and converted to grams. The USDA Food Composition Table (FCT) was used to calculate and interpret the energy and nutrient content of each food item. The estimated intake of total polyphenol and subclasses was based on the Phenol-Explorer database (www.phenol-explorer.eu/contents) [18].

### Measurement of covariates

The physical activity level of each participant was assessed by the Modifiable Activity Questionnaire which has previously been validated for the Iranian population [19]. A metabolic equivalent (MET-h/week) was calculated according to a list of common and daily routine activities.

Weight and height were collected to the nearest 0.1 kg and 0.1 cm, respectively. The weight was recorded in light clothing via a SECA digital weighing scale (Seca 707; Seca Corporation; range 0·1–150 kg), and height was taken without shoes on. BMI was defined as weight (kg) divided by square of height (m^2^). Arterial blood pressure was measured manually, using a mercury sphygmomanometer with a suitable cuff size for each participant after a 15-min rest.

Systolic (SBP) and Diastolic blood pressures were included the initial tapping and disappearance of Korotkoff sound, respectively. Blood pressure was measured twice and the average was considered as participant’s final measurement. Blood samples were taken from all participants at the TLGS research laboratory after a 12–14 h fasting.

Fasting plasma glucose (FPG) and 2-h plasma glucose (equivalent to 75 g anhydrous glucose; Cerestar EP) were measured by enzymatic colorimetric using glucose oxidase and with inter-and intra-assay CV < 2 %. Serum creatinine was measured by the standard colorimetric Jaffe_Kinetic reaction at baseline (2006–2008) and after 6 years of follow-up (2012–2015). Both Intra- and inter-assay CVs were below 3.1 %. All analyses were performed using commercial kits (Pars Azmoon Inc.).

### Definition

Although the morphological abnormalities of the kidneys or 3-month persistent urinalysis can distinctively define CKD, the glomerular filtration rate (eGFR) reduction is accepted as a more precise index of renal function. Therefore, in this study, the eGFR was expressed as ml/min/1.73m^2^ of body surface area, using the Modification of Diet in Renal Disease (MDRD) equation [20] as follows.

$$ \mathrm{eGFR}=186\times {\left(\mathrm{Serum}\ \mathrm{creatinine}\right)}^{-1.154}\times {\left(\mathrm{Age}\right)}^{-0.203}\times \left(0.742\ \mathrm{if}\ \mathrm{female}\right)\times \left(1.210\ \mathrm{if}\ \mathrm{African}-\mathrm{American}\right) $$

Patients were classified based on the eGFR levels pertain to the National Kidney Foundation Guidelines [2]. In this regard, eGFR ≥ 60 ml/min/1.73m^2^ was considered as non-CKD and eGFR < 60 ml/min/1.73m^2^ represented CKD diagnosis. Hypertension was primarily defined as SBP/DBP ≥ 140/90 mm-Hg or current therapy for a definite diagnosis of hypertension [21]. Diabetes was also defined in accordance with the criteria of the American Diabetes Association (ADA) as fasting plasma glucose ≥ 126 mg/dl, 2-h post 75-g glucose load ≥ 200 mg/dl or current therapy for a definite diagnosis of diabetes [22].

### Statistical analysis

In this study, the normal distribution of the variables was assessed by Kolmogorov–Smirnov test and Histogram chart. The participants’ characteristics and nutritional status across quartiles of total polyphenols were represented by mean ± SD and median [IQR] for normal and skewed distribution. Categorical variables were also reported by percentage. Linear regression model and Chi-square test were used for the trend of continuous and categorical variables in association with total polyphenol quartiles, respectively. Hazard ratio (HR) and 95 % confidence intervals (CI) of CKD incidence across the quartiles of total polyphenols were assessed by Cox regression analysis and the lowest quartile was considered as reference. Three models were specified for the analyses. The first model remained unadjusted for the variables. The second and third models were adjusted for sex, age, physical activity, total calorie intake, BMI, diabetes and hypertension. The proportionality assumption underlying the Cox model was examined, and no evidence of violation was observed. All analyses were performed via IBM SPSS version 16 (SPSS, Chicago, IL, USA) and *P* < 0.05 was considered significant (two-tailed).

## Results

In this study, the median (IQR) age of participants was 36 years (27–46) and men had a contribution of 47 % to the total study population. Within 11058.464 person-years of follow-up, 355 new cases of CKD were documented. Baseline characteristics and nutritional status of the participants across quartiles of total polyphenols are illustrated in Table 1. The data highlights the increasing trends of age (*P* for trend = 0.015), BMI (*P* for trend = 0.001), energy (*P* for trend < 0.001), and fat (*P* for trend = 0.003) across quartiles of total polyphenols, and the contradicting scenario for carbohydrates (*P* for trend < 0.001).

**Table 1:** Baseline characteristics and nutritional status of participants across quartiles of total polyphenols

	Quartiles of total polyphenol				*P* for trend
	Q1	Q2	Q3	Q4	
Median (mg/d)	737	1201	1717	2682	
Age (year)	34 [26–44]	37 [26–46]	36 [27–46]	36 [28–47]	0.015
Male (%)	24	26	25	25	0.693
Body mass index (kg/m^2^)	26.3 ± 4.6	26.3 ± 4.7	26.9 ± 4.8	27.2 ± 4.8	0.001
Current smoking (%)	24	29	24	23	0.236
Physical activity (MET/hours per week)	27.7 ± 46.5	29.9 ± 46.6	29.4 ± 51.3	31.7 ± 49.5	0.261
Diabetes (%)	27	31	13	29	0.789
Hypertension (%)	21	22	28	28	0.117
eGFR (ml/min/1.73 m^2^)	76.2 ± 9.5	75.8 ± 10.1	76.1 ± 1.0	75.5 ± 9.5	0.266
Total energy intake (Kcal)	1756 ± 553	2176 ± 601	2417 ± 631	2769 ± 673	< 0.001
Protein (% of energy)	13.5 ± 2.6	13.7 ± 2.3	13.8 ± 2.3	13.7 ± 2.3	0.371
Carbohydrate (% of energy)	56.7 ± 7.5	56.3 ± 6.9	57.1 ± 7.2	59.4 ± 6.9	< 0.001
Fat (% of energy)	31.3 ± 7.6	32.3 ± 6.8	31.7 ± 7.1	30.4 ± 6.3	0.003
Whole grains (g/1000 kcal)	21.6 ± 25.0	26.8 ± 30.1	27.0 ± 33.3	27.7 ± 31.0	0.005
Vegetables (g/1000 kcal)	91.1 ± 56.1	115.9 ± 59.8	134.8 ± 75.5	149.7 ± 100.2	< 0.001
Fruits (g/1000 kcal)	94.0 ± 66.6	132.0 ± 74.1	174.7 ± 94.4	263.3 ± 130.4	< 0.001
Nuts (g/1000 kcal)	2.6 ± 3.6	2.8 ± 3.9	3.2 ± 3.8	4.0 ± 5.4	< 0.001

Abbreviations: *eGFR *Estimated glomerular filtration rate, *MET *Metabolic equivalentData are presented as mean ± s.d for continuous variables or median [IQR] and percent for categorical variables

The HRs for total polyphenol and its subclasses are described in Table 2. There were no significant associations between the total polyphenols and the incidence of CKD (HR: 0.97, 95 % CI 0.67–1.40). Furthermore, the CKD incidence was less significant among participants in the fourth quartile of flavonoids and phenolic acids comparing to the first quartile (HR: 1.07, 95 % CI 0.74–1.55 and HR: 1.14, 95 % CI 0.79–1.64, respectively). Across the subclasses of polyphenols, lignans had significant association with CKD incidence in the multivariable-adjusted model, with 34 % and 31 % reduced risk of CKD in the second and third quartiles comparing to the reference, respectively. Also, the association between total dietary polyphenols and the incidence of CKD in the first and fourth quartiles were insignificant. Compared with the first quartile of stilbenes, the HR of the last unadjusted model was 0.64 (95 % CI 0.48–0.87), which had a significant inverse trend that disappeared after adjusting for potential confounders.

**Table 2:** Multivariable-adjusted COX regression (95 % CIs) for incidence of chronic kidney disease according to quartiles of the total polyphenols and its subgroups

	Total polyphenols Quartiles				*P* for trend
	Q1	Q2	Q3	Q4	
*Total Polyphenols*					
* Median* (mg/d)	737	1201	1717	2682	
Cases/ total	84/514	95/513	83/514	93/513	
Model 1	1	1.20 (0.90–1.62)	1.00 (0.73–1.35)	1.15 (0.85–1.54)	0.615
Model 2	1	1.20 (0.87–1.66)	0.92 (0.65–1.31)	0.99 (0.69–1.43)	0.598
Model 3	1	1.12 (0.81–1.55)	0.90 (0.63–1.28)	0.97 (0.67–1.40)	0.624
*Flavonoids*					
* Median* (mg/d)	33.11	55.85	82.65	125.47	
Cases/ total	83/514	86/513	93/514	93/513	
Model 1	1	1.08 (0.80–1.47)	1.13 (0.84–1.53)	1.14 (0.85–1.53)	0.401
Model 2	1	1.13 (0.81–1.57)	1.03 (0.74–1.44)	1.05 (0.73–1.52)	0.957
Model 3	1	1.12 (0.80–1.57)	1.09 (0.78–1.52)	1.07 (0.74–1.55)	0.852
*Phenolic acids*					
* Median* (mg/d)	37.21	61.71	92.21	163.62	
Cases/ total	85/514	91/513	86/514	93/513	
Model 1	1	1.09 (0.81–1.47)	1.03 (0.76–1.39)	1.11 (0.83–1.49)	0.587
Model 2	1	1.15 (0.83–1.58)	1.14 (0.82–1.60)	1.15 (0.80–1.64)	0.583
Model 3	1	1.14 (0.82–1.58)	1.13 (0.81–1.59)	1.14 (0.79–1.64)	0.600
*Lignans*					
* Median* (mg/d)	1.14	2.65	4.98	11.13	
Cases/ total	102/514	70/513	80/514	103/513	
Model 1	1	0.63 (0.47–0.86)	0.75 (0.56- 1.00)	0.98 (0.74–1.28)	0.268
Model 2	1	0.66 (0.47–0.92)	0.70 (0.50–0.97)	1.06 (0.77–1.45)	0.089
Model 3	1	0.66 (0.47–0.94)	0.69 (0.49–0.97)	1.10 (0.80–1.52)	0.049
*Stilbens*					
* Median* (mg/d)	0.05	0.16	0.28	0.70	
Cases/ total	110/514	95/514	77/513	73/513	
Model 1	1	0.85 (0.65–1.12)	0.66 (0.49–0.88)	0.64 (0.48–0.87)	0.005
Model 2	1	1.16 (0.86–1.56)	0.99 (0.71–1.37)	1.10 (0.78–1.55)	0.779
Model 3	1	1.19 (0.87–1.62)	1.02 (0.73–1.42)	1.13 (0.80–1.61)	0.672

Model 1: CrudeModel 2: Adjusted for sex, age, physical activity, and total calorie intakeModel 3: Additionally adjusted for body mass index, diabetes, and hypertension

Further adjustment for fat, carbohydrate, whole grains, vegetables, fruits, and nuts did not have a substantial impact on the association between total polyphenol and its subclasses with CKD incidence.

## Discussion

This study investigated the association between total dietary polyphenol and its major subclasses with CKD incidence among adults in Tehran, Iran. In this regard, high intake of lignans was negatively associated with CKD incidence independent of the potential cofounders. However, no similar associations were depicted with higher values than 6.8 mg. CKD incidence had a 36 % decrease in the highest quartile of stilbenes comparing to the lowest quartile of the unadjusted model. Also, total polyphenols and the incidence of CKD did not have any significant associations. No significant associations were reported after adjusting for the confounding factors, which can be explained by the considerable effect of potential confounders.

To the best of our knowledge, this is the first study that concentrated on the association between the long-term intake of total polyphenol and its major subclasses with the CKD incidence. Results of this study confirm the CKD- protective properties of lignan at a moderate amount (≤ 6.8 mg), which can be attributed to the high content of antioxidants. It is suggested that increased antioxidant defenses and subsequently, reduced levels of oxidative stress may reduce the CVD risk factors [23]. In this context, some epidemiological studies have emphasized that CVD and CKD share some common risk factors including low serum HDL cholesterol, hypertension, hypertriglyceridemia, and hyperglycemia [21, 24]. Although the CVD risk factors tend to increase progressively as a result of renal function reduction, the proper management of the cardiovascular system, could decrease the risk of CVD and CKD manifestations [21, 24].

The existing data on the consumption of lignan remains inconsistent [25, 26]. While a moderate intake of lignin promotes beneficial health effects, excessive amounts could act as estrogen antagonists [27] or enzyme inhibitor in the metabolism of sex hormones such as 5-a-reductase and 17b-hydroxysteroid dehydrogenase [22]. In other words, higher intake of lignan may have null effects on CVD risk factors, for which two mechanisms have been proposed [22], including the increased CVD risk factors and decreased level of free estradiol and testosterone in women and men, respectively [22]. Therefore, it seems as only a moderate amount of lignan (≤ 6.8 mg) may appear effective with an approximate 32 % decrease in CVD risk factors, and subsequently, CKD.

Borges et al. has performed a study on diabetic nephropathy patients to approve the CKD-protective properties of green tee polyphenols [4]. It was suggested that decreasing albuminuria [4] and inhibition of the inflammatory mediators (such as TNF-a) may be the underlying mechanisms. This study has also indicated that total polyphenols were not associated with the lower risk of CKD [4]. Meanwhile, Cynthia et al. have reported contradicting findings that can be explained by the interaction of the multiple treatments received by the diabetic participants and the green tee polyphenols. In this sense, a meta-analysis endorsed the preliminary support of polyphenol-rich interventions in the improvement of CVD risk factors among hemodialysis patients [28]. Despite individual studies in support of significant improvements, pooled results were contradicting due to the exclusion of myeloperoxidase, diastolic blood pressure, and triglycerides from the outcomes [28]. Myeloperoxidase, as a measure of oxidative stress, was the only factor with a large pooled effect size [28]. Also, the individualized polyphenol metabolism as a result of individual gastrointestinal microbiome content, brought diversity to the range of responses among the population [28]. In this respect, no specific associations were observed in the mentioned study, which could conceal the beneficial effect of total polyphenol intake.

This study had potential limitations that must be considered, as well. Firstly, the total intake of polyphenols and its subclasses were estimated by the Phenol-Explorer database () due to the unavailability of the Iranian version. Secondly, while CKD can be defined as abnormalities in the renal structure or function that persist for more 3 months and possess health challenges, this study has merely considered eGFR reduction to address CKD. Thirdly, the albumin-to-creatinine ratio (ACR) was not assessed and a creatinine double-checking procedure did not take place, which may have affected the accuracy of the findings and the interpretations. Also, larger sample sizes may have yielded significant associations. Lastly, despite the adjustments for potential confounders, the impact of residual confounders could not be ruled out.

## Conclusions

In conclusion, the result of this prospective study did not confirm any associations between total polyphenols and the incidence of CKD. However, it was finalized that lignan can only be CKD- protective by approximately 32 %, when consumed moderately (≤ 6.8 mg) and higher amounts of lignan possess no significant effects. Therefore, there is no doubt that further research is required to differentiate the impact of low, moderate and high amounts of lignan intake on CKD incidence.

## Data Availability

The datasets used and/or analyzed during the current study are available from the corresponding author on reasonable request.

## Abbreviations

CKD: Chronic Kidney Disease
GFR: Glomerular Filtration Rate
TLGS: Tehran Lipid and Glucose Study
DASH: Dietary Approaches to Stop Hypertension
FFQ: Food-Frequency Questionnaire
FCT: Food Composition Table
MDRD: Modification of Diet in Renal Disease
RIES: Research Institute for Endocrine Sciences
TNF-a: Tumor necrosis factor-alpha
